# The impact of a ball sports combination training program on physical fitness and body mass Index in children with autism spectrum disorder

**DOI:** 10.3389/fped.2025.1590666

**Published:** 2025-07-17

**Authors:** Hai-Yong Mao, Kai Qi, Shu-Qiao Meng, Ke-Long Cai, Li-Na Zhu, Wei-Ke Zhang, Zhi-Yuan Qiao, Yang Yang, Ai-Guo Chen

**Affiliations:** ^1^ Department of Physical Education, Yangzhou University, Yangzhou, China; ^2^ Faculty of Physical Education, Gdańsk University of Physical Education and Sport, Gdans, Poland; ^3^ Department of Physical Education, Xidian University, Xian, China; ^4^ Department of Physical Education, Qixiu Middle School, Nantong, China; ^5^ Institute of Sports and Brain Health, Nanjing Institute of Physical Education, Nanjing, China

**Keywords:** ball sports, autism spectrum disorder, children, physical fitness, body mass index

## Abstract

**Objective:**

This study aims to explore the effects of a 12-week Ball Combination Training Program (BCTP) on the physical fitness and Body Mass Index (BMI) of children with Autism Spectrum Disorder (ASD).

**Methods:**

This study employed a 2 × 2 mixed experimental design. A total of 29 children diagnosed with ASD were included (mean age = 7.60 ± 2.81). The 29 participants were randomly assigned to either the BCTP group (*n* = 15) or the control group (*n* = 14). The BCTP group underwent 12 weeks of ball combination exercise training (5 times per week, 45 min per session) in addition to their regular rehabilitation therapy, while the control group continued their usual rehabilitation therapy and daily activities. BMI and physical fitness tests were conducted before and after the 12-week intervention.

**Results:**

The 12-week BCTP prevented increase in BMI (*p* < 0.05), which increased in the control group. Children in the BCTP group also showed improvements in physical fitness, which was measured using the 2 × 10-meter shuttle run, sit-and-reach, and tennis ball throw (*p* < 0.05), while no such improvements were seen in the control group.

**Conclusion:**

The 12-week BCTP prevented an increase in BMI and improved the physical fitness of children with ASD in terms of speed, agility, flexibility, and upper body strength. Therefore, we recommend incorporating ball combination exercises into the physical rehabilitation of children with ASD to improve overall physical health and fitness.

## Introduction

Autism Spectrum Disorder (ASD) is a neurodevelopmental disorder that negatively impacts an individual's social interaction, communication, and behavior patterns. Its symptoms typically appear before the age of three and persist throughout life (1, 2). According to data from the Centers for Disease Control and Prevention (CDC) in 2020, the prevalence of ASD continues to rise yearly, and affects as many as 1 in 36 children by the age of eight (3). The increasing prevalence of ASD has become a significant public health issue worldwide.

Research on the physical and mental health development of children with ASD has become a prominent topic, especially experiencing reduced physical fitness and increased Body Mass Index (BMI). Studies have shown that children with ASD often exhibit significant deficiencies in motor skills, physical coordination, and levels of physical activity (4, 5). Due to social impairments, language issues, and restricted behavioral patterns, children with ASD often lack interaction and physical activity with peers, which can delayed development of physical fitness. The lack of physical fitness further exacerbates their social deficits (6). Compared to neurotypically developing children, children with ASD exhibit decreased physical activity due to their sedentary behavior, social impairments, heightened sensitivity to environmental changes, and limited interests (7, 8). Significantly impairing their weight management and physical development. Reports indicate that children with ASD are at a much higher risk of obesity compared to their peers (9), with a risk approximately 30% higher than typically developing children and 50% higher than children with other developmental disabilities or physical impairments (10, 11). Excessive weight gain will increase the risk of obesity-related diseases typically occurring in adulthood, such as diabetes and cardiovascular diseases (12). Therefore, there is an urgent need to develop strategies for improving the physical fitness of children with ASD to reducing the risk of developing obesity and preventing its associated health complications.

The introduction of sports as a rehabilitation modality has become increasingly applied in the care of children with ASD population due to its advantage of low cost, ease of implementation, and absence of side effects. For example, exercise programs such as learning motor skills (i.e., running, jumping, kicking), dance, and power cycling have been beneficial (13–15). Sports programs that use balls as the medium of activity demonstrate unique advantages, showing more significant effects in improving physical fitness-related indicators (16–18). However, most current research tends to focus on a single type of activity, controlling for unrelated variables to explore its impact on children with ASD (19). This is challenging, due to limited interests of children with ASD who may struggle to maintain interest in a single activity and often switch focus quickly (20). Thus, single-sport programs can suffer from difficulty sustaining interest and low engagement, which ultimately diminishes the effectiveness of exercise in improving the physical condition of children with ASD (21).

A more targeted intervention approach is needed particularly as each sport involves specialized movements that can yield different physical benefits. For example, single-leg dribbling exercises in soccer can effectively improve balance and lower body strength (22), while dribbling and shooting techniques in Mini-Basketball Training Program (MBTP) focus more on the development of upper body strength and speed (16). We hypothesized that combining mini-basketball and soccer into a Ball Combination Training Program (BCTP) may provide greater benefit than either sport alone.

To the best of our knowledge, most existing studies have primarily focused on the core symptoms of ASD (i.e., social impairments and repetitive behaviors). Moreover, no study has yet explored the use of a ball combination approach for the physical rehabilitation of ASD. Therefore, this study aims to investigate the effectiveness of a 12-week BCTP intervention in improving physical fitness and limiting BMI in children with ASD. This study proposes two hypotheses: (1) BCTP can effectively reduce the BMI of children with ASD. (2) BCTP can effectively improve the physical fitness of children with ASD.

## Methods

### Study design

This study employed a 2 × 2 mixed experimental design, with two groups (experimental and control) as the between-subjects factor and time (baseline and post-test) as the within-subjects factor. Eligible children were assigned to either the BCTP group or the control group. Before the intervention began, detailed demographic data for each child were collected. Additionally, parent-reported data on the CSHQ, CEBQ, and physical fitness test results assessed by the evaluators were gathered.

### Ethics

This study was approved by the Ethics and Human Protection Committee of the Affiliated Hospital of Yangzhou University and registered with the Chinese Clinical Trial Registry (ChiCTR2400087262). Informed consent was obtained from the parents or guardians of all participants. The study adhered to the ethical standards outlined in the Declaration of Helsinki.

### Participants

**Figure 1:**
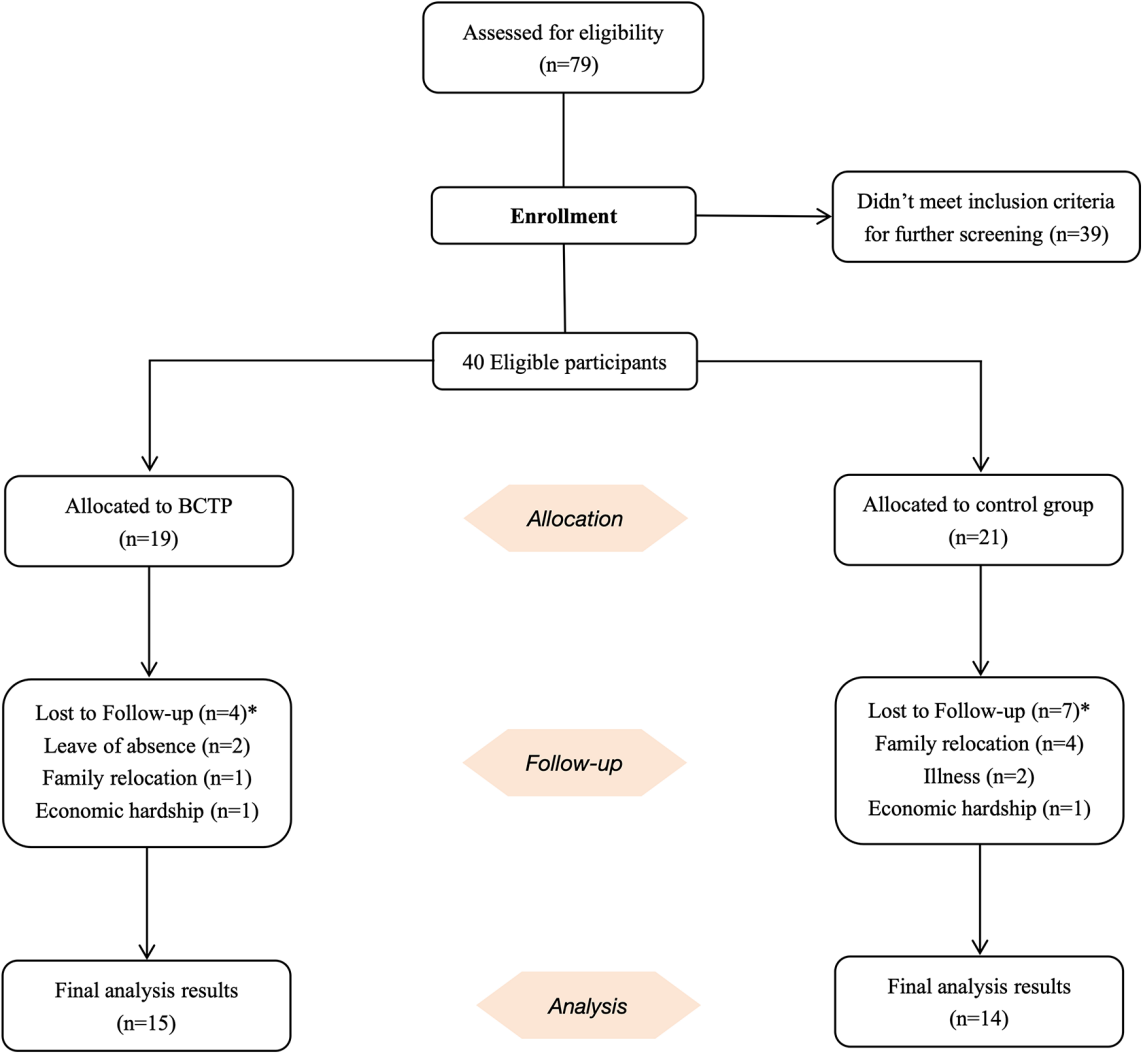
Flow diagram of study participants. *11 children did not complete the post-test assessment.

A total of 79 children from a child development center in Yangzhou expressed a willingness to participate. Participants were excluded if they met any of the following criteria: (1) participation in basketball training, soccer training, or regular physical exercise within the past 6 months; (2) the presence of comorbid psychiatric disorders; (3) complex neurological disorders (e.g., epilepsy, phenylketonuria, fragile X syndrome, tuberous sclerosis); (4) visual or auditory impairments; (5) a history of head injury; (6) any other health conditions that may limit participation in the exercise intervention. After screening, a total of 40 children with ASD took part in the experiment which included 19 children assigned to the BCTP group and 21 children assigned to the control group. In the experimental group, two children were excluded due to extended leave of absence, one dropped out because of family financial issues, and one transferred to another school due to parental job relocation. In the control group, four children lost contact after moving with their parents to other locations, two participated in other treatments due to illness, and one left due to financial difficulties. Ultimately, 29 children completed the entire study, with 11 children lost to follow-up (Figure 1).

Given the significant attrition rate, we used G*Power software to calculate the sample size to prevent potential impacts on the subsequent statistical analyses (23). Te parameter settings for this calculation were as follows: statistical test = ANOVA for repeated measures, within-between interaction; effect size *f* = 0.30; statistical power = 0.80; significance level (*α* err prob) = 0.05; correlation among repeated measures = 0.5; and number of groups = 2; number of measurements = 2. The results indicated that 12 participants in each group, for a total of 24, would meet the requirements for statistical analysis. Therefore, the number of participants included in this study satisfies the statistical analysis criteria.

### Intervention program

**Figure 2:**
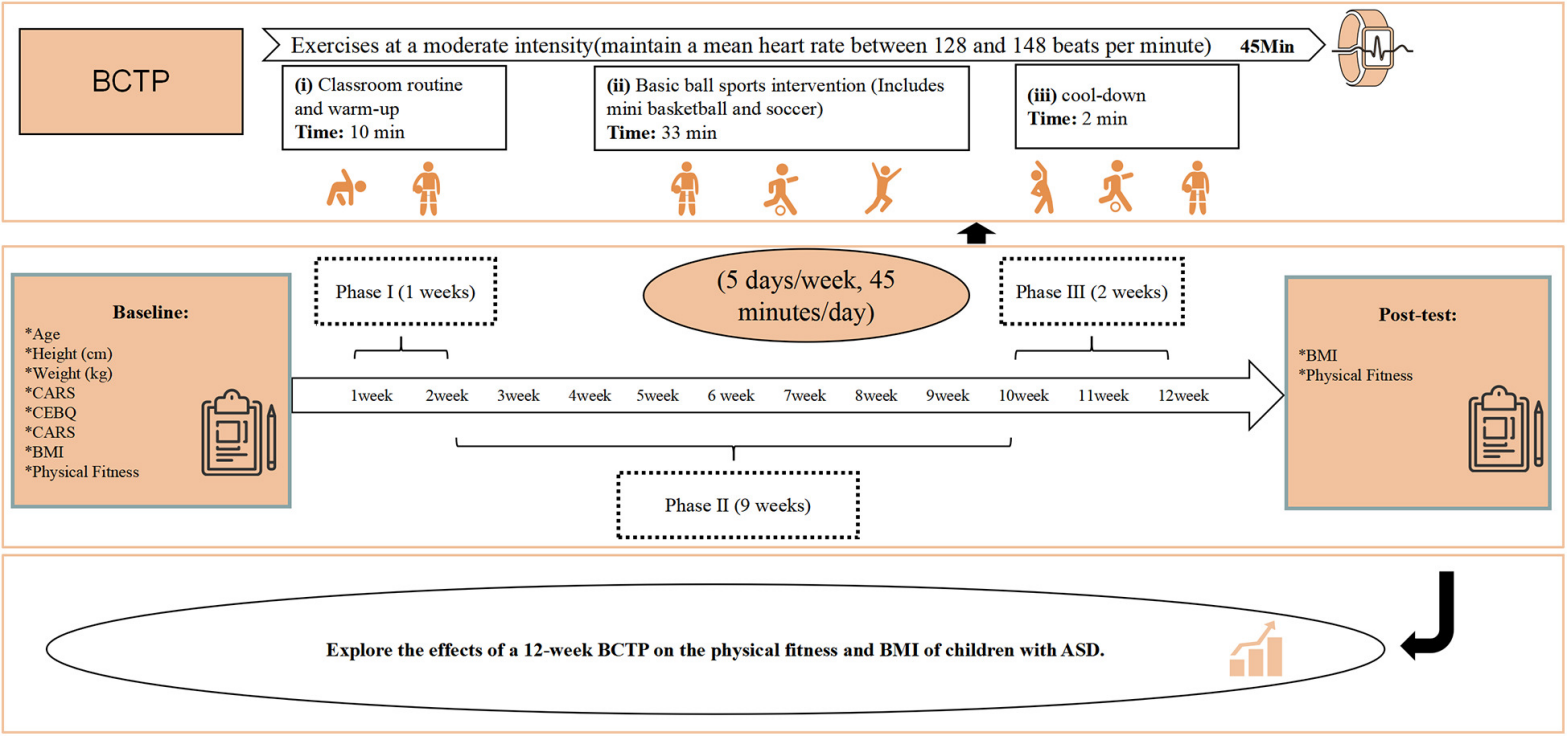
The 12-week BCTP intervention program.

All participants received regular rehabilitation treatment at the institution (ABA, TEACH, Floor Time, RDI), with the experimental group also receiving BCTP 5 times a week in 45 min sessions, for a total of 12 weeks. The details of the single-session intervention program are provided in Supplementary Appendix 1. The training content was implemented in three phases and the full training details for the BCTP group is outlined in Supplementary Appendix 2. The exercise intensity was moderate (16) and monitored using a POLAR M430 heart rate monitor to maintain the children's average heart rate between 128 and 148 beats per minute. The overall intervention program is shown in Figure 2.

The entire intervention program was conducted by two professional physical education teachers, with at least one parent accompanying each participant throughout the sessions. To ensure the course progress and intervention effectiveness, attendance was monitored, with a continuous absence not exceeding three days and a cumulative total of no more than seven days. The results from the 12-week assessment showed that during the intervention, the experimental group's average heart rate was 136.21 ± 6.19 beats per minute, maintaining a moderate intensity. In addition, The intervention also included healthcare provider visits every two weeks.

### Evaluation procedures

#### Body mass index (BMI)

Height and weight were measured using a calibrated standard health measuring instrument, the TM Professional 499KL Waist High Digital Scale with Height Rod (24). Height was measured with an accuracy of 0.1 cm and weight was measured with an accuracy of 0.1 kg. BMI was calculated by dividing weight (kg) by height (m) squared (kg/m^2^). For children, age-specific BMI percentiles were determined using on age-specific BMI growth charts (25). Children with a BMI percentile ≥85 were considered overweight, and those with a BMI percentile ≥95 were considered obese.

#### Physical fitness

Children's physical fitness was assessed using the *Chinese Physical Fitness Handbook* (26), which includes several evaluation items. These items involved anthropometric assessments (height and weight measurements), as well as physical fitness tests such as the 2 × 10-meter shuttle run to test speed and agility, the tennis ball throw to assess upper body strength, standing long jump to evaluate lower body strength, and the sit-and-reach test to measure flexibility. All participants were tested by professional assessors from the Autism Research Institute.

#### Fitness test descriptions

Shuttle Run: The testing area had two parallel lines drawn on the ground, spaced 10 meters apart. The participants were required to cross each line as quickly as possible using both feet. This test was performed twice, covering a total distance of 20 meters (2 × 10 meters), and the best result from the two attempts was used for further analysis.

Tennis Ball Throw: The participant stood behind a designated white line and performed the test using their dominant hand. With the arm extended upward and the elbow slightly bent, the participant threw the ball forcefully, using only upper body strength, while keeping their body straight. The test was repeated three times and the best throw (in cm) was used for analysis.

Standing Long Jump: The participant stood behind the take-off line, jumped as far forward as possible, and the distance from the take-off line to the heel of the foot closest to the line landing on the mat was measured. The test was repeated three times, with the best result (in cm) recorded for further analysis.

Sit-and-Reach: The participant sat on the floor with the assessor keeping their legs straight while the participant bent forward as far as possible, attempting to reach as far as they could. A bench with a measurement scale was placed in front of the participant, and their hands were placed on the bench. The maximum reach distance was measured. The best of two attempts was recorded for analysis (cm).

### Control of confounding variables

Demographic data (age, gender, weight, and height) were collected at baseline for all participants. The Childhood Autism Rating Scale (CARS), a standard method for evaluating the extent of ASD, was used to assess the severity of autism (27). It is known that dietary behaviors and sleep problems can also affect a child's weight and activity level (28). To ensure homogeneity among participants, dietary behaviors and sleep issues were analyzed as confounding variables. Dietary behaviors were assessed using the Children's Eating Behavior Questionnaire (CEBQ) (29), a parent-reported questionnaire. Sleep patterns were assessed using the Children's Sleep Habits Questionnaire (CSHQ) (30), a widely used tool for evaluating sleep issues in preschool-aged children with ASD that has shown high consistency (31).

### Statistical analysis

Independent samples *t*-tests and chi-square tests were first conducted using Jamovi 1.0.7.0 statistical software to assess homogeneity between the BCTP group and the control group based on demographic data (age, gender, and BMI), as well as the CARS, CSHQ, and CEBQ scores. If differences in these variables were found, they were included as covariates in subsequent analyses. A repeated measures analysis of variance (ANOVA) was then conducted to examine the effects of the 12-week exercise intervention on BMI and physical fitness in children with ASD. Partial eta-squared (*η*p^2^) was used to indicate the effect size. In cases of significant time × group interactions, follow-up simple effects analyses were performed. A *p*-value of <0.05 was considered statistically significant.

## Results

Chi-square tests showed no significant differences between the two groups in terms of gender (*χ*^2^ = 1.01, *p* > 0.05), age [*t*(27) = −1.493, *p* > 0.05], severity of autism (CARS) [*t*(27) = 0.054, *p* > 0.05], sleep habits (CSHQ) [*t*(27) = −0.086, *p* > 0.05], or dietary behaviors (CEBQ) [*t*(27) = −0.449, *p* > 0.05]. However, there were significant differences in height [*t*(27) = −3.074, *p* < 0.05] and weight [*t*(27) = −2.167, *p* < 0.05]. Therefore, height and weight were treated as covariates in the subsequent statistical analysis (Table 1). The mean and standard deviation of BMI and physical fitness scores for all children are shown in Table 2.

**Table 1:** Descriptive statistics of demographic variables and confounding variables in children with autism (M ± SD).

Variable	Control Group	BCTP Group	*P*
*N*	14	15	–
Gender (boys/girls)	13/1	12/3	0.333
Age (years)	8.70 ± 2.91	6.87 ± 2.59	0.147
Height (cm)	135.87 ± 12.22	121.33 ± 21.37	0.005*
Weight (kg)	32.79 ± 8.81	25.77 ± 8.63	0.039*
CARS	36.25 ± 7.28	36.13 ± 6.37	0.957
CSHQ	52.86 ± 9.53	52.60 ± 6.32	0.932
CEBQ	54.29 ± 13.45	52.20 ± 11.51	0.657

* *p* < 0.05.

CARS, childhood autism rating scale; CSHQ, children’s sleep habits questionnaire; CEBQ, child eating behavior questionnaire.

**Table 2:** Descriptive statistics of BMI and physical fitness (M ± SD).

Variable	Control Group (*n* = 14)		BCTP Group (*n* = 15)	
	Pretest	Posttest	Pretest	Posttest
BMI	17.67 ± 3.86	18.45 ± 3.61	17.14 ± 2.56	16.64 ± 2.48
2 × 10 m shuttle run	9.41 ± 1.74	10.32 ± 3.96	10.86 ± 2.21	8.76 ± 1.72
Sit-and-Reach	15.21 ± 7.30	14.18 ± 8.84	22.44 ± 8.90	25.87 ± 7.73
Tennis Ball Throw	417.86 ± 156.75	403.79 ± 161.48	316.00 ± 172.74	387.33 ± 213.98
Standing Long Jump	67.29 ± 34.06	61.64 ± 35.64	46.80 ± 36.84	63.40 ± 41.04

**Figure 3:**
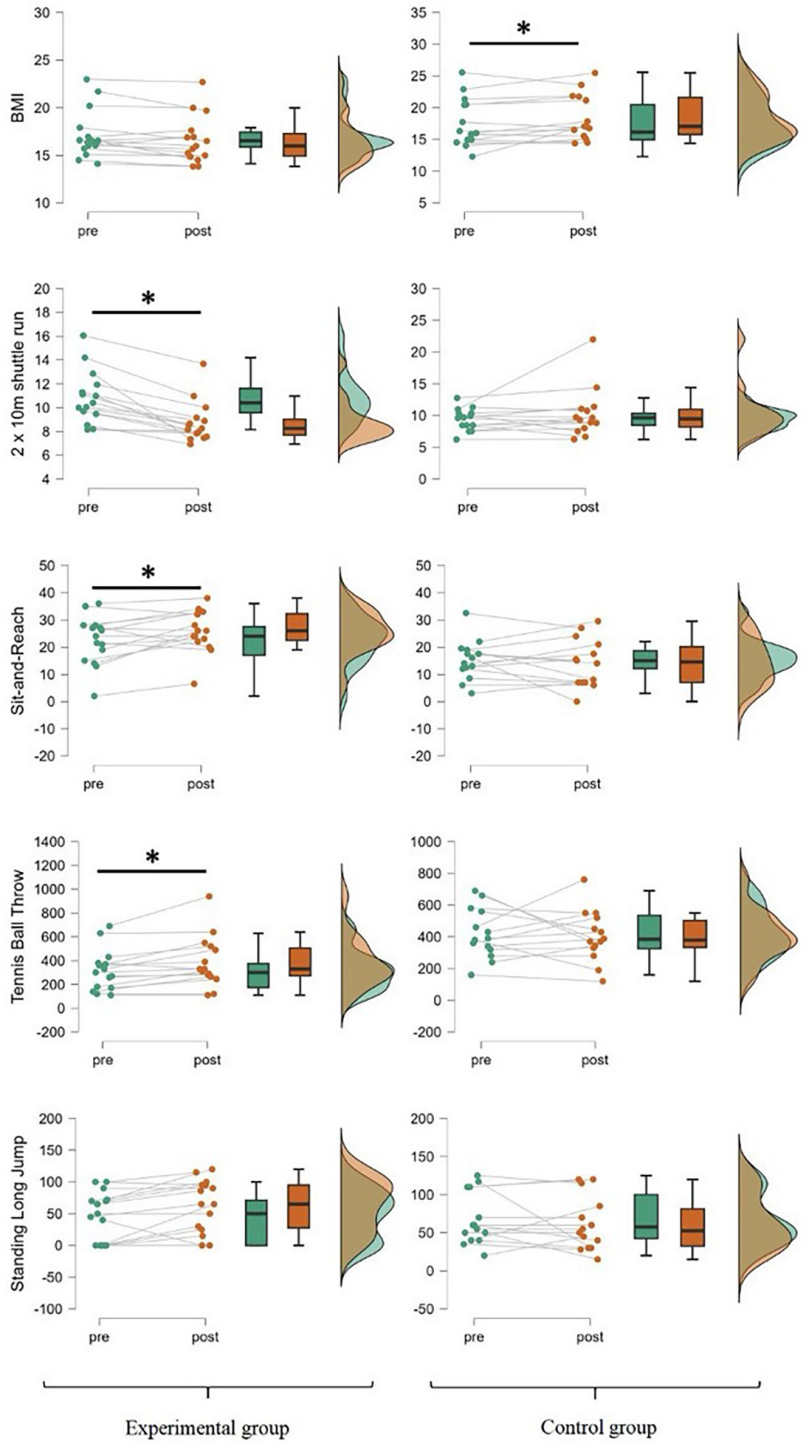
BMI values and physical fitness scores at different time points for both groups. *p < 0.05.

The analysis results of BMI and the four dimensions of physical fitness are shown in Figure 3. The study found significant time × group interaction effects on BMI [F(1, 25) = 5.390, *p* < 0.05, *η*p^2^ = 0.177], 2 × 10-meter shuttle run [F(1, 25) = 7.725, *p* < 0.05, *η*p^2^ = 0.236], sit-and-reach [F(1, 25) = 5.524, *p* < 0.05, *η*p^2^ = 0.181], and tennis ball throw [F(1, 25) = 4.573, *p* < 0.05, *η*p^2^ = 0.155]. Further simple effects analysis showed no statistically significant differences between the baseline scores of BMI, 2 × 10-meter shuttle run, sit-and-reach, and tennis ball throw in the BCTP and control groups. The BCTP group showed significant improvements in the 2 × 10-meter shuttle run, sit-and-reach, and tennis ball throw (*p* < 0.05). In contrast, the post-test BMI in the control group was significantly higher than the baseline (*p* < 0.05), suggesting a propensity for children with ASD to have a continuous increase in BMI in the absence of exercise. The results suggest that a BCTP can aid in weight management and improve the physical fitness of children with autism.

## Discussion

Numerous studies have shown that exercise can help children with autism improve their physical fitness by enhancing cardiovascular function, increasing muscle strength, and promoting physical fitness, thereby supporting healthy weight management (32). This study investigates the impact of a 12-week BCTP on the physical fitness and BMI of children with ASD. Over time, we observed positive effects in preventing weight gain and improving speed, agility, flexibility, and upper limb muscle strength. However, there was no significant improvement in lower limb muscle strength, although a trend of improvement was noted.

For analyzing BMI data, we referred to the CDC's children's BMI growth charts (25). Among the study participants, approximately 35% of the children were overweight or obese, with an age-specific BMI percentile ≥85%. Considering the heterogeneity in height and weight between the two groups of children, we included these variables as covariates to balance the group differences. After 12 weeks of a BCTP intervention, although no significant improvement was observed in the experimental group, the BMI of the control group (which did not participate in the exercise) significantly increased. A prior study analyzing the 24-hour activity composition of children with ASD predicted that increasing moderate-to-vigorous physical activity would effectively reduce weight, while increasing low-intensity exercise would have a negative impact on BMI (13). However, our exploration of body composition may be somewhat limited, as BMI is directly influenced by height and weight, there is a significant positive correlation between them (33). Encouragingly, we observed a significant reduction in BMI among all overweight and obese children in the BCTP group. Therefore, we conclude that BCTP is effective for weight management in children with ASD. Children with ASD often display stereotypical eating behaviors, which lead to a preference for high-calorie, low-nutrient foods, thereby increasing their risk of obesity (34–37). A study combining healthy eating and physical activity over the course of one year found that children with ASD experienced significant weight loss, with their BMI decreasing from the obese to the overweight category (38). Although this study did not control for diet, we still observed significant changes in the BMI of children with ASD. We anticipate that if the BCTP intervention continues for more than 12 weeks, it could lead to a significant reduction in BMI and a decrease in the incidence of overweight or obesity among children with ASD, as the various aerobic exercises in the BCTP program are effective methods for weight loss.

Study participants in the BCTP group showed significant improvements in speed, agility, and flexibility, consistent with prior reports. The gait abnormalities, poor joint flexibility, and slow movement speed commonly seen in children with ASD make it difficult for them to effectively develop physical fitness (39–41). Circular fitness exercises (including actions such as coming, walking, stopping, crawling, falling, walking, jumping, holding, leaving, sitting) helped establish basic movement skills for ASD children through verbal instructions, allowing them to complete movements independently. After 12 weeks of training, significant improvements in speed, agility, and flexibility were observed (42). Another single-sport program, a mini-basketball exercise plan, also showed positive effects on the physical fitness of children with ASD, but no significant improvements were observed in flexibility (16). However, Pan et al.'s aquatic sports program demonstrated benefits for the flexibility of children with ASD (8). This highlights that different types of sports programs may have varying effects on physical fitness in children with ASD. Unlike other single-sport programs, the BCTP in this study combined mini-basketball and soccer to incorporate ball skills, physical exercises, sports games, and stretching activities. This diverse content enriched the program, effectively improving various aspects of physical fitness, while also increasing participants' interest and engagement.

Existing evidence confirms that physical exercise helps improve muscle strength and motor performance in children with ASD (43, 44). However, in our study, we only observed improvements in upper body muscle strength, although a positive trend in lower body strength was noted, no significant changes were observed. An 8-week intervention focusing on basic motor skills effectively improved muscle activity levels in 10 children with ASD (45). Therefore, we believe this may be related to the structure of our intervention plan. The first 7 weeks of training focused primarily on mini-basketball, emphasizing upper body skills such as dribbling, passing, and shooting. In contrast, the soccer intervention, which included extensive lower-body strength training, lasted only 3 weeks. This difference may explain why no significant improvements in lower body strength were observed.

Currently, poor dietary habits (46), sleep issues (47), and sedentary behavior (48) in children with ASD limit their opportunities for physical activity, increasing the risk of overweight or obesity and significantly affecting their physical fitness. This study is the first to explore the basic physical indicators of children with ASD using a combination of ball sports. We observed several beneficial effects, yet there are still some limitations of this work. Since this is a combined intervention program, the amount of learning content increased. For children with moderate-to-severe ASD, a 12-week exercise cycle with five sessions per week might not be long enough to learn the content needed to see a measurable physical benefit. Future interventions should consider extending the intervention period or adding a single-sport group for comparison, which might be more valuable. In addition, children with autism represent a unique group characterized by significant individual differences. Apart from the relatively small sample size, there was also a high participant attrition rate, making it challenging to achieve complete randomization during group assignment, and baseline differences posed further difficulties. Since height and weight are directly related to BMI, it is recommended to interpret the findings regarding the BCTP's effect on improving BMI in children with ASD with caution. Future research should also focus on optimizing the intervention methods to align with the personalized characteristics of this population, maximizing the effectiveness of the intervention. Lastly, Due to limitations in research resources and testing conditions, we used BMI as the primary indicator. While BMI is widely used and convenient, it does not provide a comprehensive assessment of body composition. Future research should consider employing more advanced techniques, such as Bioelectrical Impedance Analysis (BIA), Dual-energy x-ray Absorptiometry (DXA), or total body water estimation, to enhance the precision and depth of physical health assessments in this population. Despite these limitations, it is clear that a BCTP can improve the physical indicators of children with ASD. We recommend incorporating a combination of different ball sports in the exercise interventions for children with ASD and developing personalized intervention plans that cater to the specific needs of children with ASD at different age stages.

## Conclusion

Our study demonstrates that BCTP intervention can improve the physical health of children with ASD and has a positive effect on weight management. BCTP intervention enriches the methods for physical rehabilitation in children with ASD, making it a promising exercise therapy. It also lays the foundation for the future diversification and personalization of exercise interventions for this population.

## Data Availability

The raw data supporting the conclusions of this article will be made available by the authors, without undue reservation.

## References

[1] American Psychiatric Association. Diagnostic and Statistical Manual of Mental Disorders. 5th ed. Washington, DC: American Psychiatric Association (2013). p. 591–643.

[2] Huang M Lin Y Wei Z Zhang S Wan G. Analysis of factors influencing the early diagnosis of autism spectrum disorder. Chin J Child Health Care. (2018) 26(04):407–11.

[3] Maenner MJ Warren Z Williams AR Amoakohene E Bakian AV Bilder DA Prevalence and characteristics of autism spectrum disorder among children aged 8 years—autism and developmental disabilities monitoring network, 11 sites, United States, 2020. MMWR Surveill Summ. (2023) 72(2):1–14. 10.15585/mmwr.ss7202a1 PMC10042614 36952288

[4] Petty S Ellis A. The meaning of autistic movements. Autism. (2024) 28(12):3015–20. 10.1177/13623613241262151 38907717 PMC11575101

[5] Martel M Finos L Bahmad S Koun E Salemme R Sonié S Motor deficits in autism differ from that of developmental coordination disorder. Autism. (2024) 28(2):415–32. 10.1177/13623613231171980 37226824

[6] Fournier KA Hass CJ Naik SK Lodha N Cauraugh JH. Motor coordination in autism spectrum disorders: a synthesis and meta-analysis. J Autism Dev Disord. (2010) 40(10):1227–40. 10.1007/s10803-010-0981-3 20195737

[7] Wang LAL Petrulla V Zampella CJ Waller R Schultz RT. Gross motor impairment and its relation to social skills in autism spectrum disorder: a systematic review and two meta-analyses. Psychol Bull. (2022) 148(3-4):273–300. 10.1037/bul0000358 35511567 PMC9894569

[8] Pan CY. The efficacy of an aquatic program on physical fitness and aquatic skills in children with and without autism spectrum disorders. Res Autism Spectr Disord. (2011) 5(1):657–65. 10.1016/j.rasd.2010.08.001

[9] Hill AP Zuckerman KE Fombonne E. Obesity and autism. Pediatrics. (2015) 136(6):1051–61. 10.1542/peds.2015-1437 26527551 PMC4657601

[10] Curtin C Anderson SE Must A Bandini L. The prevalence of obesity in children with autism: a secondary data analysis using nationally representative data from the national survey of Children's Health. BMC Pediatr. (2010) 10:11. 10.1186/1471-2431-10-11 20178579 PMC2843677

[11] Phillips KL Schieve LA Visser S Boulet S Sharma AJ Kogan MD Prevalence and impact of unhealthy weight in a national sample of US adolescents with autism and other learning and behavioral disabilities. Matern Child Health J. (2014) 18(8):1964–75. 10.1007/s10995-014-1442-y 24553796 PMC5328414

[12] De Hert M Detraux J van Winkel R Yu W Correll CU. Metabolic and cardiovascular adverse effects associated with antipsychotic drugs. Nat Rev Endocrinol. (2011) 8(2):114–26. 10.1038/nrendo.2011.156 22009159

[13] Samaritter R Payne H. Through the Kinesthetic Lens: observation of social attunement in autism Spectrum disorders. Behav Sci (Basel). (2017) 7(1):14. 10.3390/bs7010014 28335467 PMC5371758

[14] Bremer E Lloyd M. School-based fundamental-motor-skill intervention for children with- autism-like characteristics: an exploratory study. Adapt Phys Activ Q. (2016) 33(1):66–88. 10.1123/APAQ.2015-0009 26785501

[15] MacDonald M Esposito P Hauck J Jeong I Hornyak J Argento A Bicycle training for youth with down syndrome and autism spectrum disorders. Focus Autism Other Dev Disabl. (2012) 27(1):12–21. 10.1177/1088357611428333

[16] Cai K Yu Q Herold F Liu Z Wang J Zhu L Mini-basketball training program improves social communication and white matter integrity in children with autism. Brain Sci. (2020) 10(11):803. 10.3390/brainsci10110803 33142694 PMC7693206

[17] Jiang F Xu D. Study on the intervention effect of physical activities on children with ASD: a case study of Nanjing ningxin sunshine home for disabled people. Sichuan Sports Sci. (2018) 37(3):51–4.

[18] Ju X Liu H Xu J Hu B Jin Y Lu C. Effect of yoga intervention on problem behavior and motor coordination in children with autism. Behav Sci (Basel). (2024) 14(2):116. 10.3390/bs14020116 38392469 PMC10886297

[19] Xiong X. Study on the brain network mechanism of exercise intervention in improving executive function in deaf-mute children (Master’s thesis). Yangzhou University, Yangzhou (2019).

[20] Spiker MA Lin CE Van Dyke M Wood JJ. Restricted interests and anxiety in children with autism. Autism. (2012) 16(3):306–20. 10.1177/1362361311401763 21705474

[21] Grove R Roth I Hoekstra RA. The motivation for special interests in individuals with autism and controls: development and validation of the special interest motivation scale. Autism Res. (2016) 9(6):677–88. 10.1002/aur.1560 26496939

[22] Howells K Sivaratnam C Lindor E He J Hyde C McGillivray J Can a community-based football program benefit motor ability in children with autism Spectrum disorder? A pilot evaluation considering the role of social impairments. J Autism Dev Disord. (2022) 52(1):402–13. 10.1007/s10803-021-04933-w 33713242

[23] Faul F Erdfelder E Lang AG Buchner A. G* power 3: a flexible statistical power analysis program for the social, behavioral, and biomedical sciences. Behav Res Methods. (2007) 39(2):175–91. 10.3758/bf03193146 17695343

[24] Corbett BA Muscatello RA Horrocks BK Klemencic ME Tanguturi Y. Differences in body mass Index (BMI) in early adolescents with autism Spectrum disorder compared to youth with typical development. J Autism Dev Disord. (2021) 51(8):2790–9. 10.1007/s10803-020-04749-0 33051783 PMC8041918

[25] US Preventive Services Task Force, Grossman DC Bibbins-Domingo K Curry SJ Barry MJ Davidson KW Screening for obesity in children and adolescents: uS preventive services task force recommendation statement. JAMA. (2017) 317(23):2417–26. 10.1001/jama.2017.6803 28632874

[26] General Administration of Sport of China. The Handbook of Physical Fitness Measurement in China. Beijing: People’s Sports Publishing House (2003). p. 67.

[27] Schopler E Reichler RJ DeVellis RF Daly K. Toward objective classification of childhood autism: childhood autism rating scale (CARS). J Autism Dev Disord. (1980) 10(1):91–103. 10.1007/BF02408436 6927682

[28] Liu T Kelly J Davis L Zamora K. Nutrition, BMI and motor competence in children with autism Spectrum disorder. Medicina (B Aires). (2019) 55(5):135. 10.3390/medicina55050135 PMC6572175 31096637

[29] Wardle J Guthrie CA Sanderson S Rapoport L. Development of the children’s eating behaviour questionnaire. J Child Psychol Psychiatry. (2001) 42(7):963–70. 10.1111/1469-7610.00792 11693591

[30] Owens JA Spirito A McGuinn M. The children’s sleep habits questionnaire (CSHQ): psychometric properties of a survey instrument for school-aged children. Sleep. (2000) 23(8):1043–51. 10.1093/sleep/23.8.1d 11145319

[31] Goodlin-Jones BL Sitnick SL Tang K Liu J Anders TF. The children’s sleep habits questionnaire in toddlers and preschool children. J Dev Behav Pediatr. (2008) 29(2):82–8. 10.1097/dbp.0b013e318163c39a 18478627

[32] Healy S Brewer B Garcia J Daly J Patterson F. Sweat, sit, sleep: a compositional analysis of 24 h movement behaviors and body mass Index among children with autism spectrum disorder. Autism Res. (2021) 14(3):545–50. 10.1002/aur.2434 33186491

[33] Müller MJ Bosy-Westphal A. Has the BMI had its day? Int J Obes. (2025) 49(1):1–3. 10.1038/s41366-024-01643-y PMC11683000 39375530

[34] Schreck KA Williams K Smith AF. A comparison of eating behaviors between children with and without autism. J Autism Dev Disord. (2004) 34(4):433–8. 10.1023/b:jadd.0000037419.78531.86 15449518

[35] Suarez MA. Laboratory food acceptance in children with autism spectrum disorder compared with children with typical development. Am J Occup Ther. (2017) 71(6):7106220020p1–220020p6. 10.5014/ajot.2017.022152 29135429

[36] Williams KE Gibbons BG Schreck KA. Comparing selective eaters with and without developmental disabilities. J Dev Phys Disabil. (2005) 17:299–309. 10.1007/s10882-005-4387-7

[37] Evans EW Must A Anderson SE Curtin C Scampini R Maslin M Dietary patterns and body mass index in children with autism and typically developing children. Res Autism Spectr Disord. (2012) 6(1):399–405. 10.1016/j.rasd.2011.06.014 22936951 PMC3427936

[38] Nabors L Overstreet A Carnahan C Ayers K. Evaluation of a pilot healthy eating and exercise program for young adults with autism Spectrum disorder and intellectual disabilities. Adv Neurodev Disord. (2021) 5(4):413–30. 10.1007/s41252-021-00214-w 34462727 PMC8387090

[39] Casassus M Poliakoff E Gowen E Poole D Jones LA. Time perception and autistic spectrum condition: a systematic review. Autism Res. (2019) 12(10):1440–62. 10.1002/aur.2170 31336032 PMC6852160

[40] Carrigan N Allez K. Cognitive behaviour therapy for post-traumatic stress disorder in a person with an autism spectrum condition and intellectual disability: a case study. J Appl Res Intellect Disabil. (2017) 30(2):326–35. 10.1111/jar.12243 26868276

[41] Lipinski S Blanke ES Suenkel U Dziobek I. Outpatient psychotherapy for adults with high-functioning autism spectrum condition: utilization, treatment satisfaction, and preferred modifications. J Autism Dev Disord. (2019) 49(3):1154–68. 10.1007/s10803-018-3797-1 30415320

[42] Arslan E Ince G Akyüz M. Effects of a 12-week structured circuit exercise program on physical fitness levels of children with autism spectrum condition and typically developing children. Int J Dev Disabil. (2020) 68(4):500–10. 10.1080/20473869.2020.1819943 35937176 PMC9351571

[43] Jia M Zhang J Pan J Hu F Zhu Z. Benefits of exercise for children and adolescents with autism spectrum disorder: a systematic review and meta-analysis. Front Psychiatry. (2024) 15:1462601. 10.3389/fpsyt.2024.1462601 39435130 PMC11491325

[44] Hilton CL Cumpata K Klohr C Gaetke S Artner A Johnson H Effects of exergaming on executive function and motor skills in children with autism spectrum disorder: a pilot study. Am J Occup Ther. (2014) 68(1):57–65. 10.5014/ajot.2014.008664 24367956

[45] Castaño PRL Suárez DPM González ER Robledo-Castro C Hederich-Martínez C Cadena HPG Effects of physical exercise on gross motor skills in children with autism spectrum disorder. J Autism Dev Disord. (2024) 54(8):2816–25. 10.1007/s10803-023-06031-5 37410256

[46] Doreswamy S Bashir A Guarecuco JE Lahori S Baig A Narra LR Effects of diet, nutrition, and exercise in children -with autism and autism spectrum disorder: a literature review. Cureus. (2020) 12(12):e12222. 10.7759/cureus.12222 33489626 PMC7815266

[47] Brand S Jossen S Holsboer-Trachsler E Pühse U Gerber M. Impact of aerobic exercise on sleep and motor skills in children with autism spectrum disorders—a pilot study. Neuropsychiatr Dis Treat. (2015) 11:1911–20. 10.2147/NDT.S85650 26346856 PMC4531010

[48] Thomas S Barnett LM Papadopoulos N Lander N McGillivray J Rinehart N. How do physical activity and sedentary behaviour affect motor competence in children with autism Spectrum disorder compared to typically developing children: a pilot study. J Autism Dev Disord. (2022) 52(8):3443–55. 10.1007/s10803-021-05205-3 34351537

