# Supplementary material for: The impact of a ball sports combination training program on physical fitness and body mass Index in children with autism spectrum disorder
Source: Front Pediatr. 2025 Jul 17;13:1590666. doi: 10.3389/fped.2025.1590666 (PMC12310629; doi:10.3389/fped.2025.1590666)
Supplement: Supplementary file 1 [file Supplementaryfile1.docx]

| **Table 1.** Single-Session Ball Combination Training Program (BCTP) Intervention and Schedule. | | | |
| --- | --- | --- | --- |
| **Session** | **Content** | **Objective** | **Time** |
| Opening | Line up, greet the teacher, attendance check | Establish classroom discipline, adapt to the classroom environment | 2 minutes |
| Warm-up | Brisk walking, jogging, stretching exercises, partner stretching, or games | Warm-up, enhance communication between teacher, students, and parents | 8 minutes |
| Exercise Intervention | Adaptation Phase: Basic ball exercises  Fundamental Phase: Learning basic skills of mini basketball and soccer  Advanced Phase: Sports games based on mini basketball and soccer | Skill learning, physical fitness practice | 33 minutes |
| Cool-down | Relaxation exercises and summary | Relaxation, social interaction, summarize and review class content, encourage and praise students | 2 minutes |
