# Supplementary material for: The impact of a ball sports combination training program on physical fitness and body mass Index in children with autism spectrum disorder
Source: Front Pediatr. 2025 Jul 17;13:1590666. doi: 10.3389/fped.2025.1590666 (PMC12310629; doi:10.3389/fped.2025.1590666)
Supplement: Supplementary file 2 [file Supplementaryfile2.docx]

| **Table 2.** 12-Week Ball Combination Training Program (BCTP) Content. | | | | |
| --- | --- | --- | --- | --- |
| **Phase** | **Week** | **Frequency** | **Duration (minutes)** | **Main Activities** |
| Adaptation Phase | Week 1 | 5 | 45 | 1. Cultivate social etiquette 2. Develop classroom routines 3. Visual and tactile desensitization exercises 4. Simple movement exercises 5. Physical fitness exercises |
| Fundamental Phase | Week 2 | 5 | 45 | 1. Children and parents cooperate in ground rolling passes 2. Children and parents cooperate in stationary chest passes 3. Children and parents cooperate in bounce passes |
|  | Week 3 | 5 | 45 | 1. Pair cooperation in ground rolling passes 2. Pair cooperation in bounce passes 3. Pair cooperation in stationary chest passes |
|  | Week 4 | 5 | 45 | 1. Trio cooperation in ground rolling passes 2. Trio cooperation in bounce passes 3. Trio cooperation in stationary chest passes |
|  | Week 5 | 5 | 45 | 1. Stationary two-handed dribbling 2. Stationary one-handed dribbling 3. Stationary alternating two-handed dribbling |
|  | Week 6 | 5 | 45 | 1. Dribbling while moving with two hands 2. Dribbling while moving with one hand 3. Alternating two-handed dribbling while moving |
|  | Week 7 | 5 | 45 | 1. Stationary two-handed chest shooting 2. Stationary dribbling and two-handed chest shooting 3. Dribbling while moving with shooting |
|  | Week 8 | 5 | 45 | 1. Alternating football tapping 2. Inside football control 3. Kicking stationary balls 4. Inside foot dribbling |
|  | Week 9 | 5 | 45 | 1. Review stationary ball kicking 2. Review alternating foot tapping 3. Review inside foot dribbling |
|  | Week 10 | 5 | 45 | 1. Review dribbling while moving 2. Review stationary ball kicking 3. Review inside foot dribbling |
| Advanced Phase | Week 11 | 5 | 45 | Ball games (incorporating mini basketball and soccer skills in exercises like dribbling relays, small-circle shooting, large-circle jumping) |
|  | Week 12 | 5 | 45 | Ball games (incorporating mini basketball and soccer skills in exercises like dribbling relays, small-circle shooting, large-circle jumping) |
